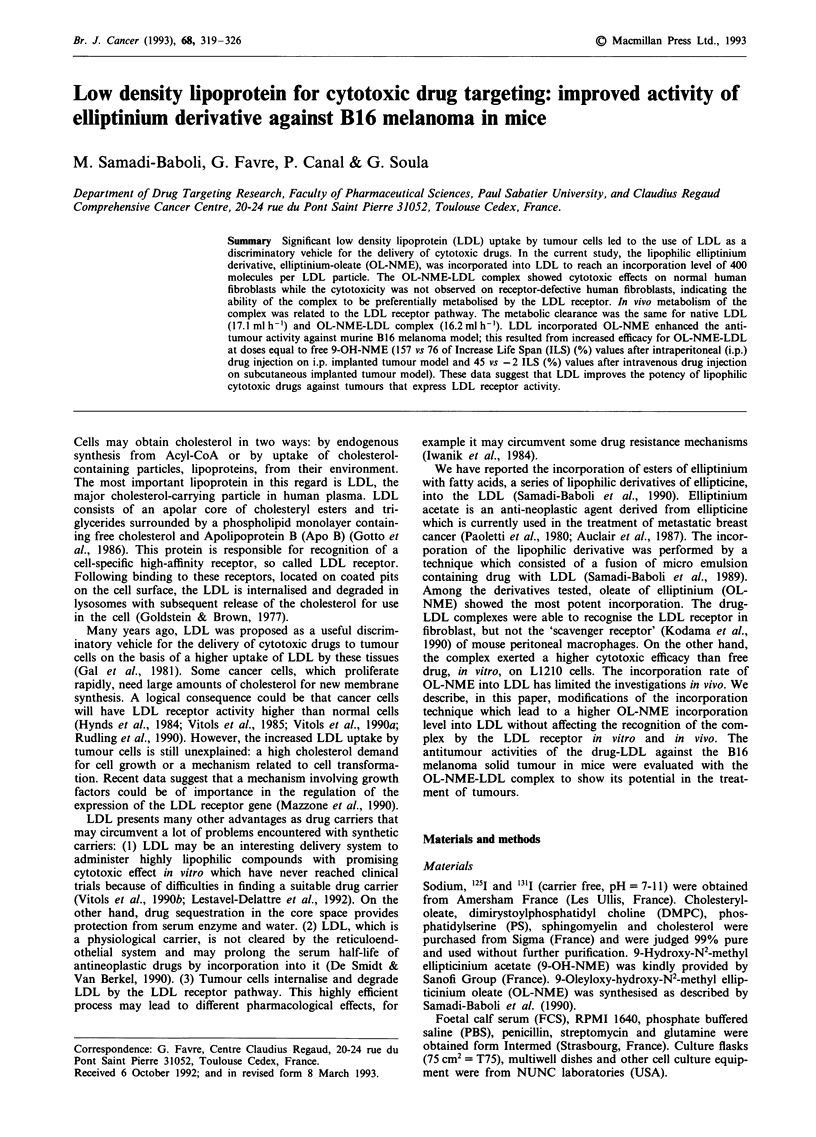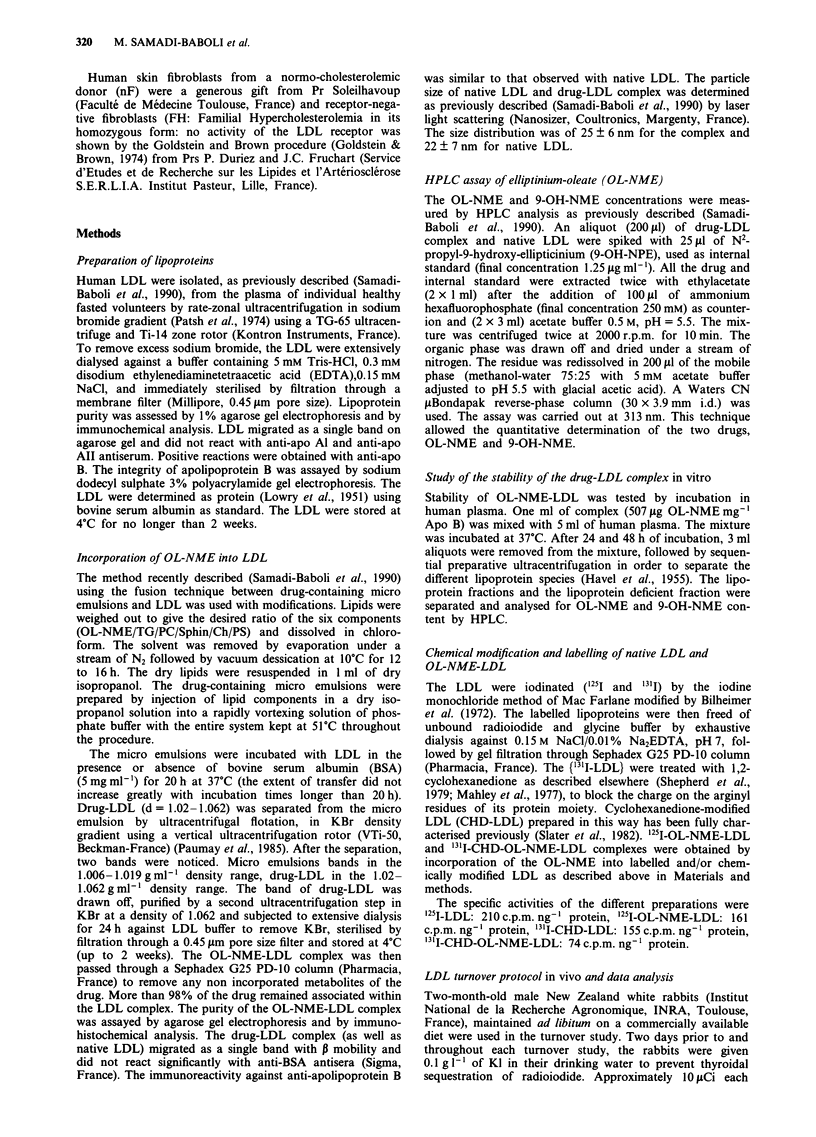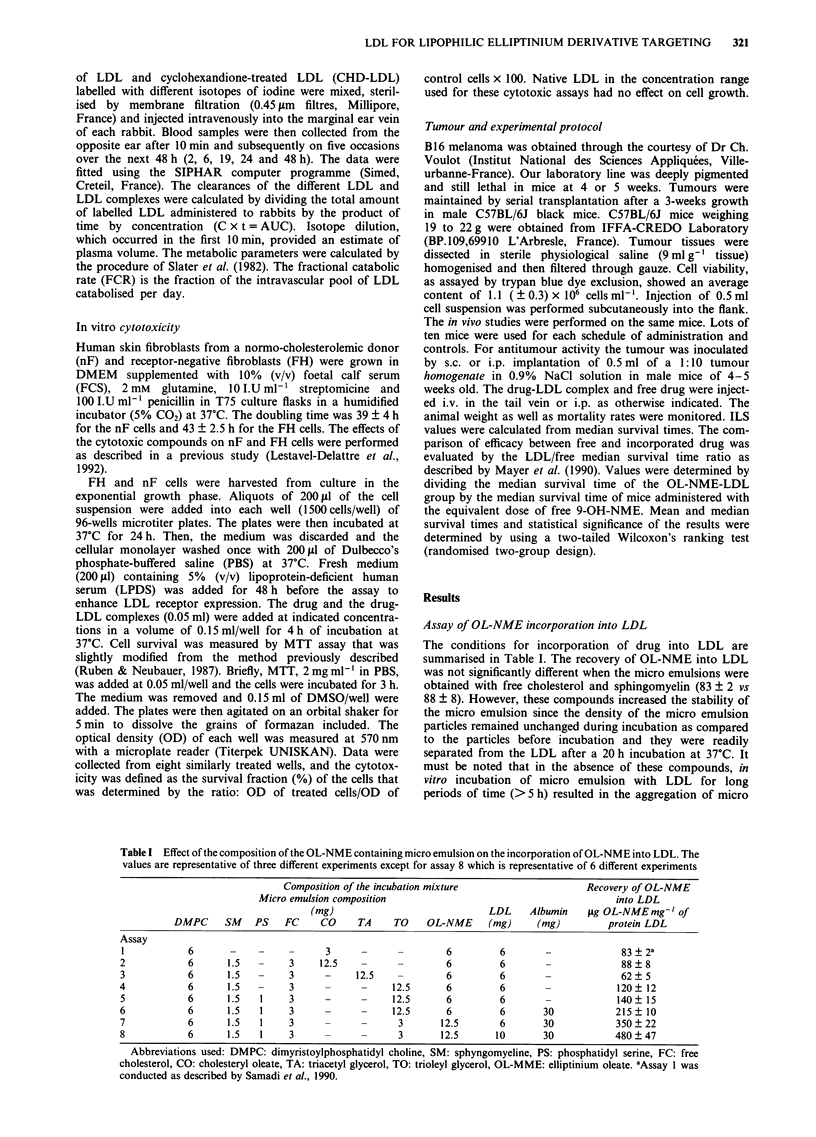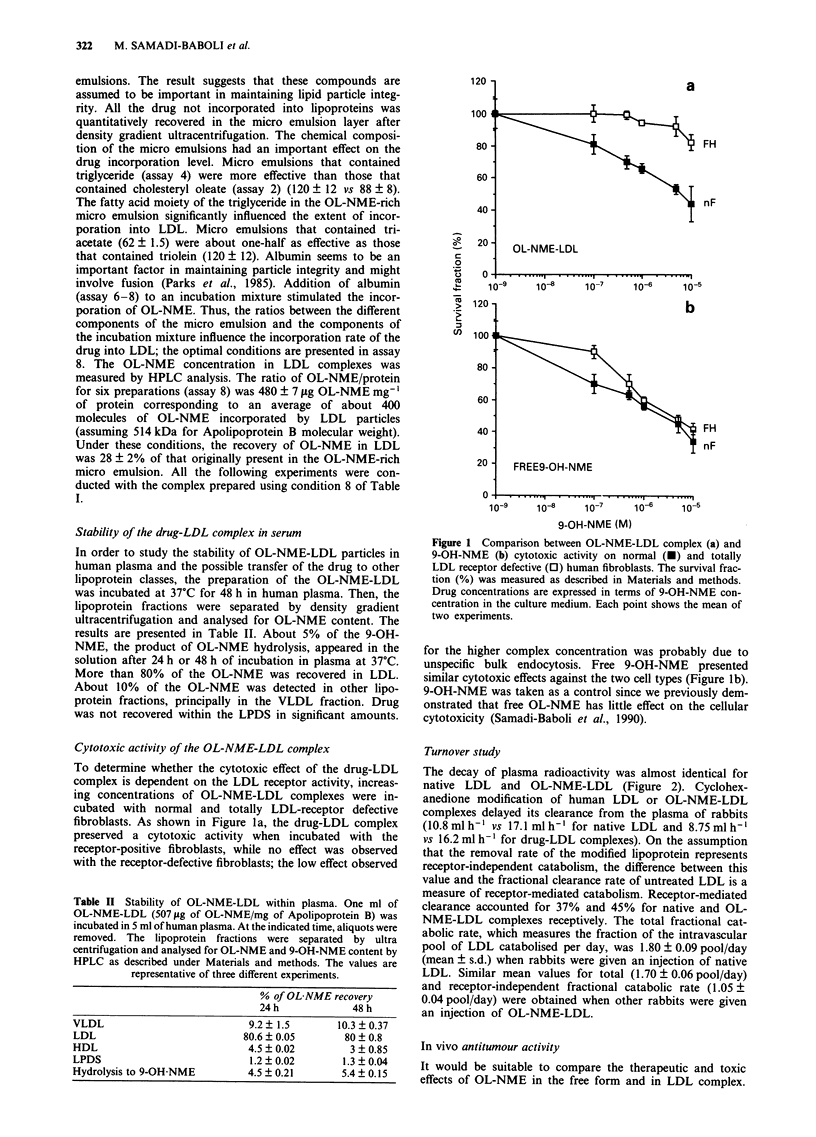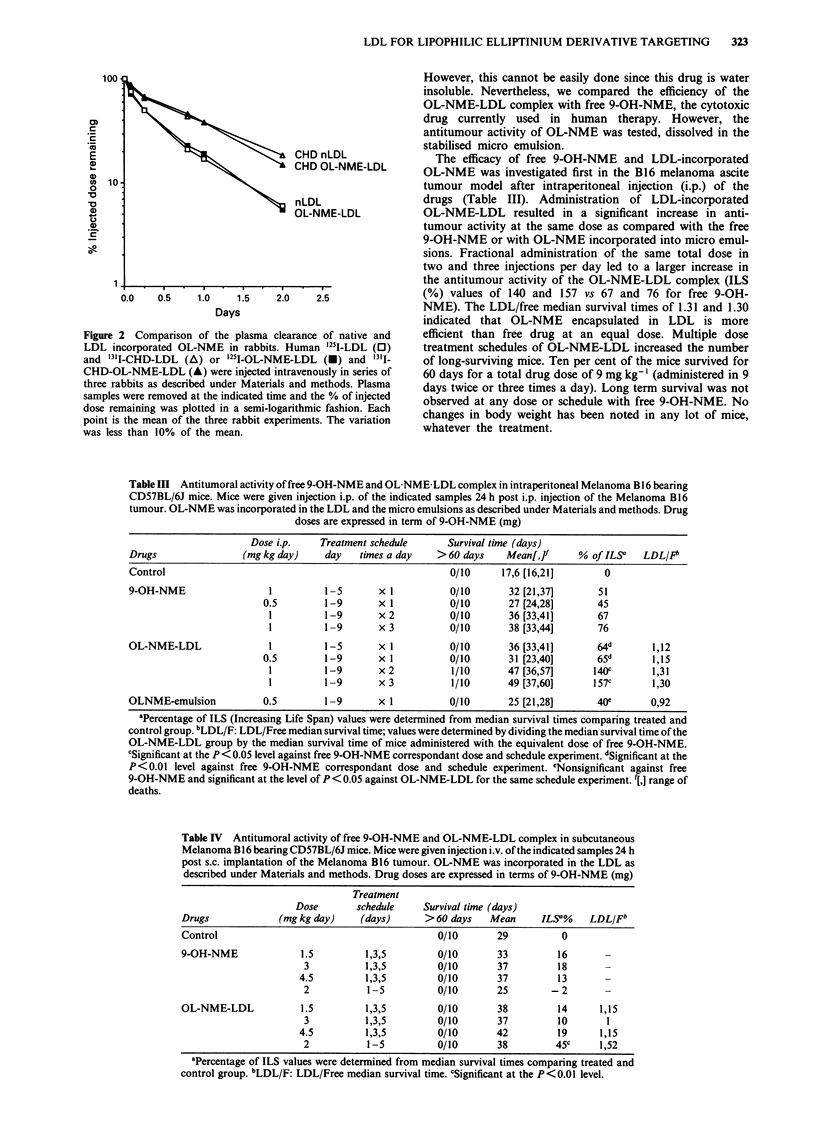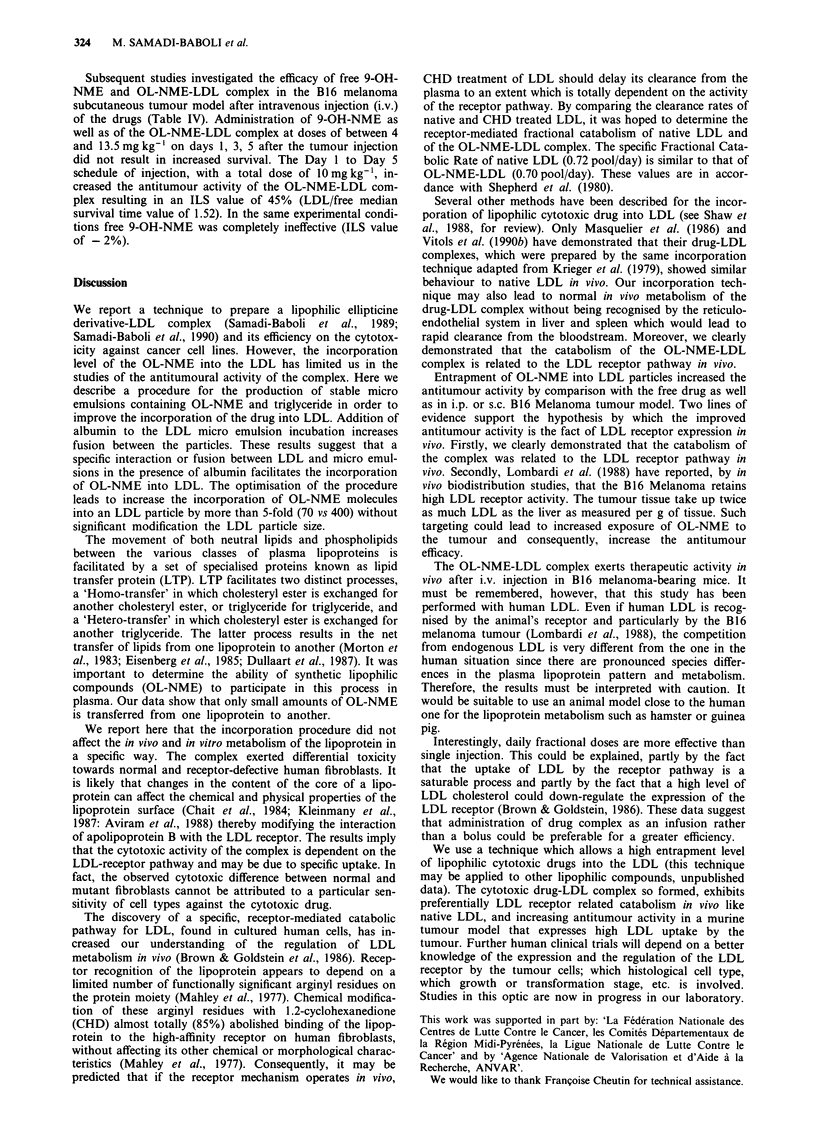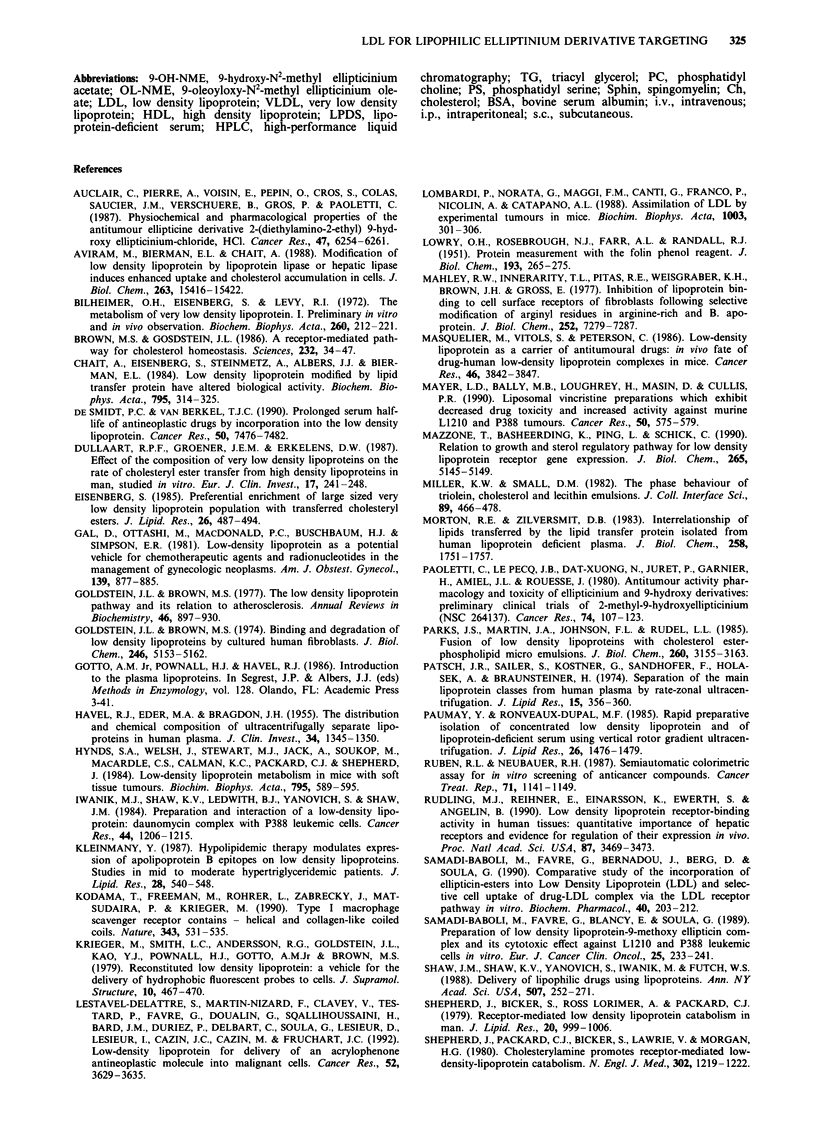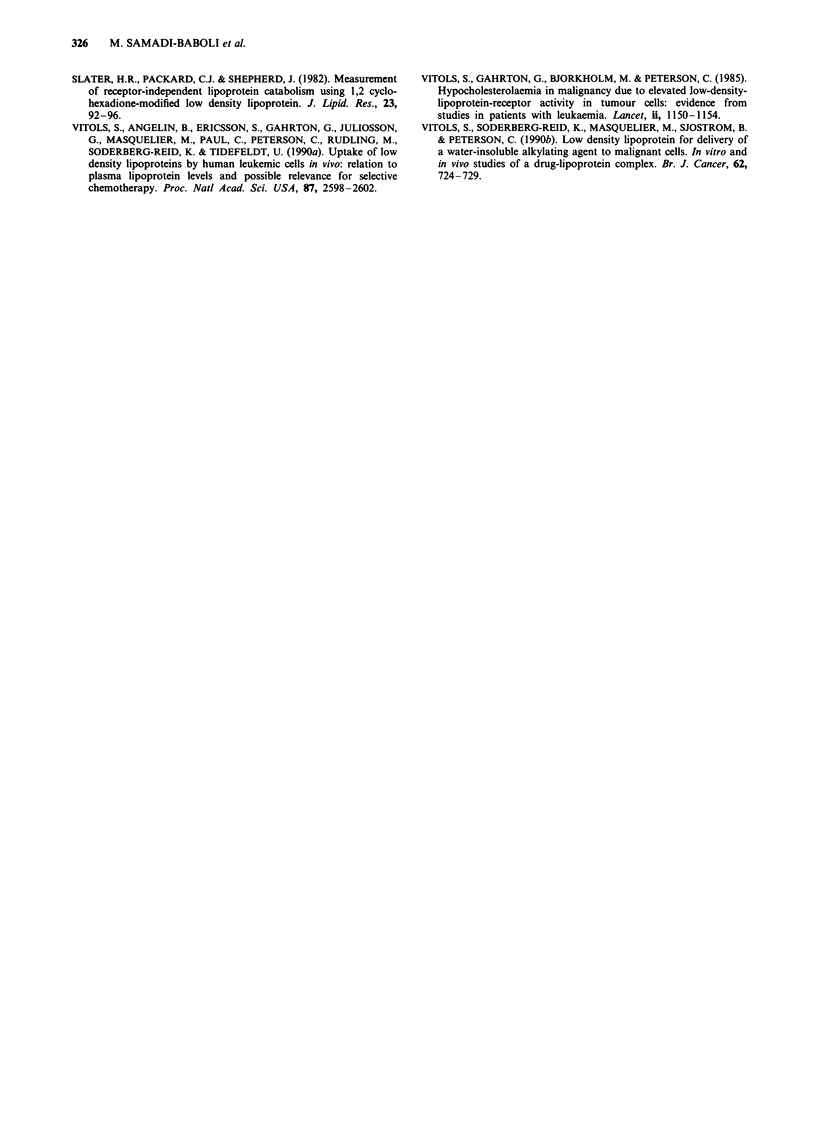# Low density lipoprotein for cytotoxic drug targeting: improved activity of elliptinium derivative against B16 melanoma in mice.

**DOI:** 10.1038/bjc.1993.335

**Published:** 1993-08

**Authors:** M. Samadi-Baboli, G. Favre, P. Canal, G. Soula

**Affiliations:** Department of Drug Targeting Research, Faculty of Pharmaceutical Sciences, Paul Sabatier University, Toulouse, France.

## Abstract

Significant low density lipoprotein (LDL) uptake by tumour cells led to the use of LDL as a discriminatory vehicle for the delivery of cytotoxic drugs. In the current study, the lipophilic elliptinium derivative, elliptinium-oleate (OL-NME), was incorporated into LDL to reach an incorporation level of 400 molecules per LDL particle. The OL-NME-LDL complex showed cytotoxic effects on normal human fibroblasts while the cytotoxicity was not observed on receptor-defective human fibroblasts, indicating the ability of the complex to be preferentially metabolised by the LDL receptor. In vivo metabolism of the complex was related to the LDL receptor pathway. The metabolic clearance was the same for native LDL (17.1 ml h-1) and OL-NME-LDL complex (16.2 ml h-1). LDL incorporated OL-NME enhanced the anti-tumour activity against murine B16 melanoma model; this resulted from increased efficacy for OL-NME-LDL at doses equal to free 9-OH-NME (157 vs 76 of Increase Life Span (ILS) (%) values after intraperitoneal (i.p.) drug injection on i.p. implanted tumour model and 45 vs -2 ILS (%) values after intravenous drug injection on subcutaneous implanted tumour model). These data suggest that LDL improves the potency of lipophilic cytotoxic drugs against tumours that express LDL receptor activity.


*Br. J. Cancer* (1993), **68**, 319–326 © Macmillan Press Ltd., 1993

### Low density lipoprotein for cytotoxic drug targeting: improved activity of elliptinium derivative against B16 melanoma in mice

M. Samadi-Baboli, G. Favre, P. Canal & G. Soula

*Department of Drug Targeting Research, Faculty of Pharmaceutical Sciences, Paul Sabatier University, and Claudius Regaud Comprehensive Cancer Centre, 20-24 rue du Pont Saint Pierre 31052, Toulouse Cedex, France.*

**Summary** Significant low density lipoprotein (LDL) uptake by tumour cells led to the use of LDL as a discriminatory vehicle for the delivery of cytotoxic drugs. In the current study, the lipophilic elliptinium derivative, elliptinium-oleate (OL-NME), was incorporated into LDL to reach an incorporation level of 400 molecules per LDL particle. The OL-NME-LDL complex showed cytotoxic effects on normal human fibroblasts while the cytotoxicity was not observed on receptor-defective human fibroblasts, indicating the ability of the complex to be preferentially metabolised by the LDL receptor. *In vivo* metabolism of the complex was related to the LDL receptor pathway. The metabolic clearance was the same for native LDL (17.1 ml $h^{-1}$) and OL-NME-LDL complex (16.2 ml $h^{-1}$). LDL incorporated OL-NME enhanced the anti-tumour activity against murine B16 melanoma model; this resulted from increased efficacy for OL-NME-LDL at doses equal to free 9-OH-NME (157 *vs* 76 of Increase Life Span (ILS) (%) values after intraperitoneal (i.p.) drug injection on i.p. implanted tumour model and 45 *vs* −2 ILS (%) values after intravenous drug injection on subcutaneous implanted tumour model). These data suggest that LDL improves the potency of lipophilic cytotoxic drugs against tumours that express LDL receptor activity.

Cells may obtain cholesterol in two ways: by endogenous synthesis from Acyl-CoA or by uptake of cholesterol-containing particles, lipoproteins, from their environment. The most important lipoprotein in this regard is LDL, the major cholesterol-carrying particle in human plasma. LDL consists of an apolar core of cholesteryl esters and triglycerides surrounded by a phospholipid monolayer containing free cholesterol and Apolipoprotein B (Apo B) (Gotto *et al.*, 1986). This protein is responsible for recognition of a cell-specific high-affinity receptor, so called LDL receptor. Following binding to these receptors, located on coated pits on the cell surface, the LDL is internalised and degraded in lysosomes with subsequent release of the cholesterol for use in the cell (Goldstein & Brown, 1977).

Many years ago, LDL was proposed as a useful discriminatory vehicle for the delivery of cytotoxic drugs to tumour cells on the basis of a higher uptake of LDL by these tissues (Gal *et al.*, 1981). Some cancer cells, which proliferate rapidly, need large amounts of cholesterol for new membrane synthesis. A logical consequence could be that cancer cells will have LDL receptor activity higher than normal cells (Hynds *et al.*, 1984; Vitols *et al.*, 1985; Vitols *et al.*, 1990*a*; Rudling *et al.*, 1990). However, the increased LDL uptake by tumour cells is still unexplained: a high cholesterol demand for cell growth or a mechanism related to cell transformation. Recent data suggest that a mechanism involving growth factors could be of importance in the regulation of the expression of the LDL receptor gene (Mazzone *et al.*, 1990).

LDL presents many other advantages as drug carriers that may circumvent a lot of problems encountered with synthetic carriers: (1) LDL may be an interesting delivery system to administer highly lipophilic compounds with promising cytotoxic effect *in vitro* which have never reached clinical trials because of difficulties in finding a suitable drug carrier (Vitols *et al.*, 1990*b*; Lestavel-Delattre *et al.*, 1992). On the other hand, drug sequestration in the core space provides protection from serum enzyme and water. (2) LDL, which is a physiological carrier, is not cleared by the reticuloendothelial system and may prolong the serum half-life of antineoplastic drugs by incorporation into it (De Smidt & Van Berkel, 1990). (3) Tumour cells internalise and degrade LDL by the LDL receptor pathway. This highly efficient process may lead to different pharmacological effects, for example it may circumvent some drug resistance mechanisms (Iwanik *et al.*, 1984).

We have reported the incorporation of esters of elliptinium with fatty acids, a series of lipophilic derivatives of ellipticine, into the LDL (Samadi-Baboli *et al.*, 1990). Elliptinium acetate is an anti-neoplastic agent derived from ellipticine which is currently used in the treatment of metastatic breast cancer (Paoletti *et al.*, 1980; Auclair *et al.*, 1987). The incorporation of the lipophilic derivative was performed by a technique which consisted of a fusion of micro emulsion containing drug with LDL (Samadi-Baboli *et al.*, 1989). Among the derivatives tested, oleate of elliptinium (OL-NME) showed the most potent incorporation. The drug-LDL complexes were able to recognise the LDL receptor in fibroblast, but not the 'scavenger receptor' (Kodama *et al.*, 1990) of mouse peritoneal macrophages. On the other hand, the complex exerted a higher cytotoxic efficacy than free drug, *in vitro*, on L1210 cells. The incorporation rate of OL-NME into LDL has limited the investigations *in vivo*. We describe, in this paper, modifications of the incorporation technique which lead to a higher OL-NME incorporation level into LDL without affecting the recognition of the complex by the LDL receptor *in vitro* and *in vivo*. The antitumour activities of the drug-LDL against the B16 melanoma solid tumour in mice were evaluated with the OL-NME-LDL complex to show its potential in the treatment of tumours.

### Materials and methods

### *Materials*

Sodium, $^{125}I$ and $^{131}I$ (carrier free, pH = 7-11) were obtained from Amersham France (Les Ullis, France). Cholesteryl-oleate, dimirystoylphosphatidyl choline (DMPC), phosphatidylserine (PS), sphingomyelin and cholesterol were purchased from Sigma (France) and were judged 99% pure and used without further purification. 9-Hydroxy-$N^2$-methyl ellipticinium acetate (9-OH-NME) was kindly provided by Sanofi Group (France). 9-Oleyloxy-hydroxy-$N^2$-methyl ellipticinium oleate (OL-NME) was synthesised as described by Samadi-Baboli *et al.* (1990).

Foetal calf serum (FCS), RPMI 1640, phosphate buffered saline (PBS), penicillin, streptomycin and glutamine were obtained form Intermed (Strasbourg, France). Culture flasks ($75\ cm^2$ = T75), multiwell dishes and other cell culture equipment were from NUNC laboratories (USA).

Correspondence: G. Favre, Centre Claudius Regaud, 20-24 rue du Pont Saint Pierre 31052, Toulouse Cedex, France.
Received 6 October 1992; and in revised form 8 March 1993.

Human skin fibroblasts from a normo-cholesterolemic donor (nF) were a generous gift from Pr Soleilhavoup (Faculté de Médecine Toulouse, France) and receptor-negative fibroblasts (FH: Familial Hypercholesterolemia in its homozygous form: no activity of the LDL receptor was shown by the Goldstein and Brown procedure (Goldstein & Brown, 1974) from Prs P. Duriez and J.C. Fruchart (Service d'Etudes et de Recherche sur les Lipides et l'Artériosclérose S.E.R.L.I.A. Institut Pasteur, Lille, France).

### Methods

### *Preparation of lipoproteins*

Human LDL were isolated, as previously described (Samadi-Baboli *et al.*, 1990), from the plasma of individual healthy fasted volunteers by rate-zonal ultracentrifugation in sodium bromide gradient (Patsh *et al.*, 1974) using a TG-65 ultracentrifuge and Ti-14 zone rotor (Kontron Instruments, France). To remove excess sodium bromide, the LDL were extensively dialysed against a buffer containing 5 mM Tris-HCl, 0.3 mM disodium ethylenediaminetetraacetic acid (EDTA),0.15 mM NaCl, and immediately sterilised by filtration through a membrane filter (Millipore, 0.45 µm pore size). Lipoprotein purity was assessed by 1% agarose gel electrophoresis and by immunochemical analysis. LDL migrated as a single band on agarose gel and did not react with anti-apo AI and anti-apo AII antiserum. Positive reactions were obtained with anti-apo B. The integrity of apolipoprotein B was assayed by sodium dodecyl sulphate 3% polyacrylamide gel electrophoresis. The LDL were determined as protein (Lowry *et al.*, 1951) using bovine serum albumin as standard. The LDL were stored at 4°C for no longer than 2 weeks.

### *Incorporation of OL-NME into LDL*

The method recently described (Samadi-Baboli *et al.*, 1990) using the fusion technique between drug-containing micro emulsions and LDL was used with modifications. Lipids were weighed out to give the desired ratio of the six components (OL-NME/TG/PC/Sphin/Ch/PS) and dissolved in chloroform. The solvent was removed by evaporation under a stream of $N_2$ followed by vacuum dessication at 10°C for 12 to 16 h. The dry lipids were resuspended in 1 ml of dry isopropanol. The drug-containing micro emulsions were prepared by injection of lipid components in a dry isopropanol solution into a rapidly vortexing solution of phosphate buffer with the entire system kept at 51°C throughout the procedure.

The micro emulsions were incubated with LDL in the presence or absence of bovine serum albumin (BSA) (5 mg ml$^{-1}$) for 20 h at 37°C (the extent of transfer did not increase greatly with incubation times longer than 20 h). Drug-LDL (d = 1.02–1.062) was separated from the micro emulsion by ultracentrifugal flotation, in KBr density gradient using a vertical ultracentrifugation rotor (VTi-50, Beckman-France) (Paumay *et al.*, 1985). After the separation, two bands were noticed. Micro emulsions bands in the 1.006–1.019 g ml$^{-1}$ density range, drug-LDL in the 1.02–1.062 g ml$^{-1}$ density range. The band of drug-LDL was drawn off, purified by a second ultracentrifugation step in KBr at a density of 1.062 and subjected to extensive dialysis for 24 h against LDL buffer to remove KBr, sterilised by filtration through a 0.45 µm pore size filter and stored at 4°C (up to 2 weeks). The OL-NME-LDL complex was then passed through a Sephadex G25 PD-10 column (Pharmacia, France) to remove any non incorporated metabolites of the drug. More than 98% of the drug remained associated within the LDL complex. The purity of the OL-NME-LDL complex was assayed by agarose gel electrophoresis and by immunohistochemical analysis. The drug-LDL complex (as well as native LDL) migrated as a single band with β mobility and did not react significantly with anti-BSA antisera (Sigma, France). The immunoreactivity against anti-apolipoprotein B was similar to that observed with native LDL. The particle size of native LDL and drug-LDL complex was determined as previously described (Samadi-Baboli *et al.*, 1990) by laser light scattering (Nanosizer, Coultronics, Margenty, France). The size distribution was of 25 ± 6 nm for the complex and 22 ± 7 nm for native LDL.

### *HPLC assay of elliptinium-oleate (OL-NME)*

The OL-NME and 9-OH-NME concentrations were measured by HPLC analysis as previously described (Samadi-Baboli *et al.*, 1990). An aliquot (200 µl) of drug-LDL complex and native LDL were spiked with 25 µl of N$^2$-propyl-9-hydroxy-ellipticinium (9-OH-NPE), used as internal standard (final concentration 1.25 µg ml$^{-1}$). All the drug and internal standard were extracted twice with ethylacetate (2 × 1 ml) after the addition of 100 µl of ammonium hexafluorophosphate (final concentration 250 mM) as counterion and (2 × 3 ml) acetate buffer 0.5 M, pH = 5.5. The mixture was centrifuged twice at 2000 r.p.m. for 10 min. The organic phase was drawn off and dried under a stream of nitrogen. The residue was redissolved in 200 µl of the mobile phase (methanol-water 75:25 with 5 mM acetate buffer adjusted to pH 5.5 with glacial acetic acid). A Waters CN µBondapak reverse-phase column (30 × 3.9 mm i.d.) was used. The assay was carried out at 313 nm. This technique allowed the quantitative determination of the two drugs, OL-NME and 9-OH-NME.

### *Study of the stability of the drug-LDL complex* in vitro

Stability of OL-NME-LDL was tested by incubation in human plasma. One ml of complex (507 µg OL-NME mg$^{-1}$ Apo B) was mixed with 5 ml of human plasma. The mixture was incubated at 37°C. After 24 and 48 h of incubation, 3 ml aliquots were removed from the mixture, followed by sequential preparative ultracentrifugation in order to separate the different lipoprotein species (Havel *et al.*, 1955). The lipoprotein fractions and the lipoprotein deficient fraction were separated and analysed for OL-NME and 9-OH-NME content by HPLC.

### *Chemical modification and labelling of native LDL and OL-NME-LDL*

The LDL were iodinated ($^{125}$I and $^{131}$I) by the iodine monochloride method of Mac Farlane modified by Bilheimer *et al.* (1972). The labelled lipoproteins were then freed of unbound radioiodide and glycine buffer by exhaustive dialysis against 0.15 M NaCl/0.01% $Na_2EDTA$, pH 7, followed by gel filtration through Sephadex G25 PD-10 column (Pharmacia, France). The {$^{131}$I-LDL} were treated with 1,2-cyclohexanedione as described elsewhere (Shepherd *et al.*, 1979; Mahley *et al.*, 1977), to block the charge on the arginyl residues of its protein moiety. Cyclohexanedione-modified LDL (CHD-LDL) prepared in this way has been fully characterised previously (Slater *et al.*, 1982). $^{125}$I-OL-NME-LDL and $^{131}$I-CHD-OL-NME-LDL complexes were obtained by incorporation of the OL-NME into labelled and/or chemically modified LDL as described above in Materials and methods.

The specific activities of the different preparations were $^{125}$I-LDL: 210 c.p.m. ng$^{-1}$ protein, $^{125}$I-OL-NME-LDL: 161 c.p.m. ng$^{-1}$ protein, $^{131}$I-CHD-LDL: 155 c.p.m. ng$^{-1}$ protein, $^{131}$I-CHD-OL-NME-LDL: 74 c.p.m. ng$^{-1}$ protein.

### *LDL turnover protocol* in vivo *and data analysis*

Two-month-old male New Zealand white rabbits (Institut National de la Recherche Agronomique, INRA, Toulouse, France), maintained *ad libitum* on a commercially available diet were used in the turnover study. Two days prior to and throughout each turnover study, the rabbits were given 0.1 g l$^{-1}$ of KI in their drinking water to prevent thyroidal sequestration of radioiodide. Approximately 10 µCi each of LDL and cyclohexandione-treated LDL (CHD-LDL) labelled with different isotopes of iodine were mixed, sterilised by membrane filtration (0.45 µm filtres, Millipore, France) and injected intravenously into the marginal ear vein of each rabbit. Blood samples were then collected from the opposite ear after 10 min and subsequently on five occasions over the next 48 h (2, 6, 19, 24 and 48 h). The data were fitted using the SIPHAR computer programme (Simed, Creteil, France). The clearances of the different LDL and LDL complexes were calculated by dividing the total amount of labelled LDL administered to rabbits by the product of time by concentration (C × t = AUC). Isotope dilution, which occurred in the first 10 min, provided an estimate of plasma volume. The metabolic parameters were calculated by the procedure of Slater *et al.* (1982). The fractional catabolic rate (FCR) is the fraction of the intravascular pool of LDL catabolised per day.

### In vitro *cytotoxicity*

Human skin fibroblasts from a normo-cholesterolemic donor (nF) and receptor-negative fibroblasts (FH) were grown in DMEM supplemented with 10% (v/v) foetal calf serum (FCS), 2 mM glutamine, 10 I.U $ml^{-1}$ streptomicine and 100 I.U $ml^{-1}$ penicillin in T75 culture flasks in a humidified incubator (5% $CO_2$) at 37°C. The doubling time was 39 ± 4 h for the nF cells and 43 ± 2.5 h for the FH cells. The effects of the cytotoxic compounds on nF and FH cells were performed as described in a previous study (Lestavel-Delattre *et al.*, 1992).

FH and nF cells were harvested from culture in the exponential growth phase. Aliquots of 200 µl of the cell suspension were added into each well (1500 cells/well) of 96-wells microtiter plates. The plates were then incubated at 37°C for 24 h. Then, the medium was discarded and the cellular monolayer washed once with 200 µl of Dulbecco's phosphate-buffered saline (PBS) at 37°C. Fresh medium (200 µl) containing 5% (v/v) lipoprotein-deficient human serum (LPDS) was added for 48 h before the assay to enhance LDL receptor expression. The drug and the drug-LDL complexes (0.05 ml) were added at indicated concentrations in a volume of 0.15 ml/well for 4 h of incubation at 37°C. Cell survival was measured by MTT assay that was slightly modified from the method previously described (Ruben & Neubauer, 1987). Briefly, MTT, 2 mg $ml^{-1}$ in PBS, was added at 0.05 ml/well and the cells were incubated for 3 h. The medium was removed and 0.15 ml of DMSO/well were added. The plates were then agitated on an orbital shaker for 5 min to dissolve the grains of formazan included. The optical density (OD) of each well was measured at 570 nm with a microplate reader (Titerpek UNISKAN). Data were collected from eight similarly treated wells, and the cytotoxicity was defined as the survival fraction (%) of the cells that was determined by the ratio: OD of treated cells/OD of control cells × 100. Native LDL in the concentration range used for these cytotoxic assays had no effect on cell growth.

### *Tumour and experimental protocol*

B16 melanoma was obtained through the courtesy of Dr Ch. Voulot (Institut National des Sciences Appliquées, Villeurbanne-France). Our laboratory line was deeply pigmented and still lethal in mice at 4 or 5 weeks. Tumours were maintained by serial transplantation after a 3-weeks growth in male C57BL/6J black mice. C57BL/6J mice weighing 19 to 22 g were obtained from IFFA-CREDO Laboratory (BP.109,69910 L'Arbresle, France). Tumour tissues were dissected in sterile physiological saline (9 ml $g^{-1}$ tissue) homogenised and then filtered through gauze. Cell viability, as assayed by trypan blue dye exclusion, showed an average content of 1.1 (± 0.3) × $10^6$ cells $ml^{-1}$. Injection of 0.5 ml cell suspension was performed subcutaneously into the flank. The *in vivo* studies were performed on the same mice. Lots of ten mice were used for each schedule of administration and controls. For antitumour activity the tumour was inoculated by s.c. or i.p. implantation of 0.5 ml of a 1:10 tumour *homogenate* in 0.9% NaCl solution in male mice of 4–5 weeks old. The drug-LDL complex and free drug were injected i.v. in the tail vein or i.p. as otherwise indicated. The animal weight as well as mortality rates were monitored. ILS values were calculated from median survival times. The comparison of efficacy between free and incorporated drug was evaluated by the LDL/free median survival time ratio as described by Mayer *et al.* (1990). Values were determined by dividing the median survival time of the OL-NME-LDL group by the median survival time of mice administered with the equivalent dose of free 9-OH-NME. Mean and median survival times and statistical significance of the results were determined by using a two-tailed Wilcoxon's ranking test (randomised two-group design).

### Results

### *Assay of OL-NME incorporation into LDL*

The conditions for incorporation of drug into LDL are summarised in Table I. The recovery of OL-NME into LDL was not significantly different when the micro emulsions were obtained with free cholesterol and sphingomyelin (83 ± 2 *vs* 88 ± 8). However, these compounds increased the stability of the micro emulsion since the density of the micro emulsion particles remained unchanged during incubation as compared to the particles before incubation and they were readily separated from the LDL after a 20 h incubation at 37°C. It must be noted that in the absence of these compounds, *in vitro* incubation of micro emulsion with LDL for long periods of time (> 5 h) resulted in the aggregation of micro

**Table I** Effect of the composition of the OL-NME containing micro emulsion on the incorporation of OL-NME into LDL. The values are representative of three different experiments except for assay 8 which is representative of 6 different experiments

| | *Composition of the incubation mixture* | | | | | | | | | | *Recovery of OL-NME into LDL* |
|---|---|---|---|---|---|---|---|---|---|---|---|
| | *Micro emulsion composition (mg)* | | | | | | | | | | |
| | *DMPC* | *SM* | *PS* | *FC* | *CO* | *TA* | *TO* | *OL-NME* | *LDL (mg)* | *Albumin (mg)* | *µg OL-NME $mg^{-1}$ of protein LDL* |
| Assay | | | | | | | | | | | |
| 1 | 6 | – | – | – | 3 | – | – | 6 | 6 | – | 83 ± 2[a] |
| 2 | 6 | 1.5 | – | 3 | 12.5 | – | – | 6 | 6 | – | 88 ± 8 |
| 3 | 6 | 1.5 | – | 3 | – | 12.5 | – | 6 | 6 | – | 62 ± 5 |
| 4 | 6 | 1.5 | – | 3 | – | – | 12.5 | 6 | 6 | – | 120 ± 12 |
| 5 | 6 | 1.5 | 1 | 3 | – | – | 12.5 | 6 | 6 | – | 140 ± 15 |
| 6 | 6 | 1.5 | 1 | 3 | – | – | 12.5 | 6 | 6 | 30 | 215 ± 10 |
| 7 | 6 | 1.5 | 1 | 3 | – | – | 3 | 12.5 | 6 | 30 | 350 ± 22 |
| 8 | 6 | 1.5 | 1 | 3 | – | – | 3 | 12.5 | 10 | 30 | 480 ± 47 |

Abbreviations used: DMPC: dimyristoylphosphatidyl choline, SM: sphyngomyeline, PS: phosphatidyl serine, FC: free cholesterol, CO: cholesteryl oleate, TA: triacetyl glycerol, TO: trioleyl glycerol, OL-MME: elliptinium oleate. [a]Assay 1 was conducted as described by Samadi *et al.*, 1990.

emulsions. The result suggests that these compounds are assumed to be important in maintaining lipid particle integrity. All the drug not incorporated into lipoproteins was quantitatively recovered in the micro emulsion layer after density gradient ultracentrifugation. The chemical composition of the micro emulsions had an important effect on the drug incorporation level. Micro emulsions that contained triglyceride (assay 4) were more effective than those that contained cholesteryl oleate (assay 2) (120 ± 12 *vs* 88 ± 8). The fatty acid moiety of the triglyceride in the OL-NME-rich micro emulsion significantly influenced the extent of incorporation into LDL. Micro emulsions that contained triacetate (62 ± 1.5) were about one-half as effective as those that contained triolein (120 ± 12). Albumin seems to be an important factor in maintaining particle integrity and might involve fusion (Parks *et al.*, 1985). Addition of albumin (assay 6–8) to an incubation mixture stimulated the incorporation of OL-NME. Thus, the ratios between the different components of the micro emulsion and the components of the incubation mixture influence the incorporation rate of the drug into LDL; the optimal conditions are presented in assay 8. The OL-NME concentration in LDL complexes was measured by HPLC analysis. The ratio of OL-NME/protein for six preparations (assay 8) was 480 ± 7 μg OL-NME $mg^{-1}$ of protein corresponding to an average of about 400 molecules of OL-NME incorporated by LDL particles (assuming 514 kDa for Apolipoprotein B molecular weight). Under these conditions, the recovery of OL-NME in LDL was 28 ± 2% of that originally present in the OL-NME-rich micro emulsion. All the following experiments were conducted with the complex prepared using condition 8 of Table I.

### *Stability of the drug-LDL complex in serum*

In order to study the stability of OL-NME-LDL particles in human plasma and the possible transfer of the drug to other lipoprotein classes, the preparation of the OL-NME-LDL was incubated at 37°C for 48 h in human plasma. Then, the lipoprotein fractions were separated by density gradient ultracentrifugation and analysed for OL-NME content. The results are presented in Table II. About 5% of the 9-OH-NME, the product of OL-NME hydrolysis, appeared in the solution after 24 h or 48 h of incubation in plasma at 37°C. More than 80% of the OL-NME was recovered in LDL. About 10% of the OL-NME was detected in other lipoprotein fractions, principally in the VLDL fraction. Drug was not recovered within the LPDS in significant amounts.

### *Cytotoxic activity of the OL-NME-LDL complex*

To determine whether the cytotoxic effect of the drug-LDL complex is dependent on the LDL receptor activity, increasing concentrations of OL-NME-LDL complexes were incubated with normal and totally LDL-receptor defective fibroblasts. As shown in Figure 1a, the drug-LDL complex preserved a cytotoxic activity when incubated with the receptor-positive fibroblasts, while no effect was observed with the receptor-defective fibroblasts; the low effect observed for the higher complex concentration was probably due to unspecific bulk endocytosis. Free 9-OH-NME presented similar cytotoxic effects against the two cell types (Figure 1b). 9-OH-NME was taken as a control since we previously demonstrated that free OL-NME has little effect on the cellular cytotoxicity (Samadi-Baboli *et al.*, 1990).

**Table II** Stability of OL-NME-LDL within plasma. One ml of OL-NME-LDL (507 μg of OL-NME/mg of Apolipoprotein B) was incubated in 5 ml of human plasma. At the indicated time, aliquots were removed. The lipoprotein fractions were separated by ultra centrifugation and analysed for OL-NME and 9-OH-NME content by HPLC as described under Materials and methods. The values are representative of three different experiments.

| | *% of OL·NME recovery* 24 h | 48 h |
|---|---|---|
| VLDL | 9.2 ± 1.5 | 10.3 ± 0.37 |
| LDL | 80.6 ± 0.05 | 80 ± 0.8 |
| HDL | 4.5 ± 0.02 | 3 ± 0.85 |
| LPDS | 1.2 ± 0.02 | 1.3 ± 0.04 |
| Hydrolysis to 9-OH·NME | 4.5 ± 0.21 | 5.4 ± 0.15 |

**Figure 1** Comparison between OL-NME-LDL complex (**a**) and 9-OH-NME (**b**) cytotoxic activity on normal (■) and totally LDL receptor defective (□) human fibroblasts. The survival fraction (%) was measured as described in Materials and methods. Drug concentrations are expressed in terms of 9-OH-NME concentration in the culture medium. Each point shows the mean of two experiments.

### *Turnover study*

The decay of plasma radioactivity was almost identical for native LDL and OL-NME-LDL (Figure 2). Cyclohexanedione modification of human LDL or OL-NME-LDL complexes delayed its clearance from the plasma of rabbits (10.8 ml $h^{-1}$ *vs* 17.1 ml $h^{-1}$ for native LDL and 8.75 ml $h^{-1}$ *vs* 16.2 ml $h^{-1}$ for drug-LDL complexes). On the assumption that the removal rate of the modified lipoprotein represents receptor-independent catabolism, the difference between this value and the fractional clearance rate of untreated LDL is a measure of receptor-mediated catabolism. Receptor-mediated clearance accounted for 37% and 45% for native and OL-NME-LDL complexes receptively. The total fractional catabolic rate, which measures the fraction of the intravascular pool of LDL catabolised per day, was 1.80 ± 0.09 pool/day (mean ± s.d.) when rabbits were given an injection of native LDL. Similar mean values for total (1.70 ± 0.06 pool/day) and receptor-independent fractional catabolic rate (1.05 ± 0.04 pool/day) were obtained when other rabbits were given an injection of OL-NME-LDL.

### In vivo *antitumour activity*

It would be suitable to compare the therapeutic and toxic effects of OL-NME in the free form and in LDL complex.

**Figure 2** Comparison of the plasma clearance of native and LDL incorporated OL-NME in rabbits. Human $^{125}$I-LDL (□) and $^{131}$I-CHD-LDL (Δ) or $^{125}$I-OL-NME-LDL (■) and $^{131}$I-CHD-OL-NME-LDL (▲) were injected intravenously in series of three rabbits as described under Materials and methods. Plasma samples were removed at the indicated time and the % of injected dose remaining was plotted in a semi-logarithmic fashion. Each point is the mean of the three rabbit experiments. The variation was less than 10% of the mean.

However, this cannot be easily done since this drug is water insoluble. Nevertheless, we compared the efficiency of the OL-NME-LDL complex with free 9-OH-NME, the cytotoxic drug currently used in human therapy. However, the antitumour activity of OL-NME was tested, dissolved in the stabilised micro emulsion.

The efficacy of free 9-OH-NME and LDL-incorporated OL-NME was investigated first in the B16 melanoma ascite tumour model after intraperitoneal injection (i.p.) of the drugs (Table III). Administration of LDL-incorporated OL-NME-LDL resulted in a significant increase in antitumour activity at the same dose as compared with the free 9-OH-NME or with OL-NME incorporated into micro emulsions. Fractional administration of the same total dose in two and three injections per day led to a larger increase in the antitumour activity of the OL-NME-LDL complex (ILS (%) values of 140 and 157 *vs* 67 and 76 for free 9-OH-NME). The LDL/free median survival times of 1.31 and 1.30 indicated that OL-NME encapsulated in LDL is more efficient than free drug at an equal dose. Multiple dose treatment schedules of OL-NME-LDL increased the number of long-surviving mice. Ten per cent of the mice survived for 60 days for a total drug dose of 9 mg $kg^{-1}$ (administered in 9 days twice or three times a day). Long term survival was not observed at any dose or schedule with free 9-OH-NME. No changes in body weight has been noted in any lot of mice, whatever the treatment.

**Table III** Antitumoral activity of free 9-OH-NME and OL·NME·LDL complex in intraperitoneal Melanoma B16 bearing CD57BL/6J mice. Mice were given injection i.p. of the indicated samples 24 h post i.p. injection of the Melanoma B16 tumour. OL-NME was incorporated in the LDL and the micro emulsions as described under Materials and methods. Drug doses are expressed in term of 9-OH-NME (mg)

| Drugs | Dose i.p. (mg kg day) | Treatment schedule day | times a day | Survival time (days) >60 days | Mean[,][f] | % of ILS[a] | LDL/F[b] |
|---|---|---|---|---|---|---|---|
| Control | | | | 0/10 | 17,6 [16,21] | 0 | |
| 9-OH-NME | 1 | 1–5 | × 1 | 0/10 | 32 [21,37] | 51 | |
| | 0.5 | 1–9 | × 1 | 0/10 | 27 [24,28] | 45 | |
| | 1 | 1–9 | × 2 | 0/10 | 36 [33,41] | 67 | |
| | 1 | 1–9 | × 3 | 0/10 | 38 [33,44] | 76 | |
| OL-NME-LDL | 1 | 1–5 | × 1 | 0/10 | 36 [33,41] | 64[d] | 1,12 |
| | 0.5 | 1–9 | × 1 | 0/10 | 31 [23,40] | 65[d] | 1,15 |
| | 1 | 1–9 | × 2 | 1/10 | 47 [36,57] | 140[c] | 1,31 |
| | 1 | 1–9 | × 3 | 1/10 | 49 [37,60] | 157[c] | 1,30 |
| OLNME-emulsion | 0.5 | 1–9 | × 1 | 0/10 | 25 [21,28] | 40[e] | 0,92 |

[a]Percentage of ILS (Increasing Life Span) values were determined from median survival times comparing treated and control group. [b]LDL/F: LDL/Free median survival time; values were determined by dividing the median survival time of the OL-NME-LDL group by the median survival time of mice administered with the equivalent dose of free 9-OH-NME. [c]Significant at the $P < 0.05$ level against free 9-OH-NME correspondant dose and schedule experiment. [d]Significant at the $P < 0.01$ level against free 9-OH-NME correspondant dose and schedule experiment. [e]Nonsignificant against free 9-OH-NME and significant at the level of $P < 0.05$ against OL-NME-LDL for the same schedule experiment. [f][,] range of deaths.

**Table IV** Antitumoral activity of free 9-OH-NME and OL-NME-LDL complex in subcutaneous Melanoma B16 bearing CD57BL/6J mice. Mice were given injection i.v. of the indicated samples 24 h post s.c. implantation of the Melanoma B16 tumour. OL-NME was incorporated in the LDL as described under Materials and methods. Drug doses are expressed in terms of 9-OH-NME (mg)

| Drugs | Dose (mg kg day) | Treatment schedule (days) | Survival time (days) >60 days | Mean | ILS[a]% | LDL/F[b] |
|---|---|---|---|---|---|---|
| Control | | | 0/10 | 29 | 0 | |
| 9-OH-NME | 1.5 | 1,3,5 | 0/10 | 33 | 16 | – |
| | 3 | 1,3,5 | 0/10 | 37 | 18 | – |
| | 4.5 | 1,3,5 | 0/10 | 37 | 13 | – |
| | 2 | 1–5 | 0/10 | 25 | – 2 | – |
| OL-NME-LDL | 1.5 | 1,3,5 | 0/10 | 38 | 14 | 1,15 |
| | 3 | 1,3,5 | 0/10 | 37 | 10 | 1 |
| | 4.5 | 1,3,5 | 0/10 | 42 | 19 | 1,15 |
| | 2 | 1–5 | 0/10 | 38 | 45[c] | 1,52 |

[a]Percentage of ILS values were determined from median survival times comparing treated and control group. [b]LDL/F: LDL/Free median survival time. [c]Significant at the $P < 0.01$ level.

Subsequent studies investigated the efficacy of free 9-OH-NME and OL-NME-LDL complex in the B16 melanoma subcutaneous tumour model after intravenous injection (i.v.) of the drugs (Table IV). Administration of 9-OH-NME as well as of the OL-NME-LDL complex at doses of between 4 and 13.5 mg $kg^{-1}$ on days 1, 3, 5 after the tumour injection did not result in increased survival. The Day 1 to Day 5 schedule of injection, with a total dose of 10 mg $kg^{-1}$, increased the antitumour activity of the OL-NME-LDL complex resulting in an ILS value of 45% (LDL/free median survival time value of 1.52). In the same experimental conditions free 9-OH-NME was completely ineffective (ILS value of − 2%).

### Discussion

We report a technique to prepare a lipophilic ellipticine derivative-LDL complex (Samadi-Baboli *et al.*, 1989; Samadi-Baboli *et al.*, 1990) and its efficiency on the cytotoxicity against cancer cell lines. However, the incorporation level of the OL-NME into the LDL has limited us in the studies of the antitumoural activity of the complex. Here we describe a procedure for the production of stable micro emulsions containing OL-NME and triglyceride in order to improve the incorporation of the drug into LDL. Addition of albumin to the LDL micro emulsion incubation increases fusion between the particles. These results suggest that a specific interaction or fusion between LDL and micro emulsions in the presence of albumin facilitates the incorporation of OL-NME into LDL. The optimisation of the procedure leads to increase the incorporation of OL-NME molecules into an LDL particle by more than 5-fold (70 *vs* 400) without significant modification the LDL particle size.

The movement of both neutral lipids and phospholipids between the various classes of plasma lipoproteins is facilitated by a set of specialised proteins known as lipid transfer protein (LTP). LTP facilitates two distinct processes, a 'Homo-transfer' in which cholesteryl ester is exchanged for another cholesteryl ester, or triglyceride for triglyceride, and a 'Hetero-transfer' in which cholesteryl ester is exchanged for another triglyceride. The latter process results in the net transfer of lipids from one lipoprotein to another (Morton *et al.*, 1983; Eisenberg *et al.*, 1985; Dullaart *et al.*, 1987). It was important to determine the ability of synthetic lipophilic compounds (OL-NME) to participate in this process in plasma. Our data show that only small amounts of OL-NME is transferred from one lipoprotein to another.

We report here that the incorporation procedure did not affect the *in vivo* and *in vitro* metabolism of the lipoprotein in a specific way. The complex exerted differential toxicity towards normal and receptor-defective human fibroblasts. It is likely that changes in the content of the core of a lipoprotein can affect the chemical and physical properties of the lipoprotein surface (Chait *et al.*, 1984; Kleinmany *et al.*, 1987: Aviram *et al.*, 1988) thereby modifying the interaction of apolipoprotein B with the LDL receptor. The results imply that the cytotoxic activity of the complex is dependent on the LDL-receptor pathway and may be due to specific uptake. In fact, the observed cytotoxic difference between normal and mutant fibroblasts cannot be attributed to a particular sensitivity of cell types against the cytotoxic drug.

The discovery of a specific, receptor-mediated catabolic pathway for LDL, found in cultured human cells, has increased our understanding of the regulation of LDL metabolism *in vivo* (Brown & Goldstein *et al.*, 1986). Receptor recognition of the lipoprotein appears to depend on a limited number of functionally significant arginyl residues on the protein moiety (Mahley *et al.*, 1977). Chemical modification of these arginyl residues with 1.2-cyclohexanedione (CHD) almost totally (85%) abolished binding of the lipoprotein to the high-affinity receptor on human fibroblasts, without affecting its other chemical or morphological characteristics (Mahley *et al.*, 1977). Consequently, it may be predicted that if the receptor mechanism operates *in vivo*, CHD treatment of LDL should delay its clearance from the plasma to an extent which is totally dependent on the activity of the receptor pathway. By comparing the clearance rates of native and CHD treated LDL, it was hoped to determine the receptor-mediated fractional catabolism of native LDL and of the OL-NME-LDL complex. The specific Fractional Catabolic Rate of native LDL (0.72 pool/day) is similar to that of OL-NME-LDL (0.70 pool/day). These values are in accordance with Shepherd *et al.* (1980).

Several other methods have been described for the incorporation of lipophilic cytotoxic drug into LDL (see Shaw *et al.*, 1988, for review). Only Masquelier *et al.* (1986) and Vitols *et al.* (1990*b*) have demonstrated that their drug-LDL complexes, which were prepared by the same incorporation technique adapted from Krieger *et al.* (1979), showed similar behaviour to native LDL *in vivo*. Our incorporation technique may also lead to normal *in vivo* metabolism of the drug-LDL complex without being recognised by the reticuloendothelial system in liver and spleen which would lead to rapid clearance from the bloodstream. Moreover, we clearly demonstrated that the catabolism of the OL-NME-LDL complex is related to the LDL receptor pathway *in vivo*.

Entrapment of OL-NME into LDL particles increased the antitumour activity by comparison with the free drug as well as in i.p. or s.c. B16 Melanoma tumour model. Two lines of evidence support the hypothesis by which the improved antitumour activity is the fact of LDL receptor expression *in vivo*. Firstly, we clearly demonstrated that the catabolism of the complex was related to the LDL receptor pathway *in vivo*. Secondly, Lombardi *et al.* (1988) have reported, by *in vivo* biodistribution studies, that the B16 Melanoma retains high LDL receptor activity. The tumour tissue take up twice as much LDL as the liver as measured per g of tissue. Such targeting could lead to increased exposure of OL-NME to the tumour and consequently, increase the antitumour efficacy.

The OL-NME-LDL complex exerts therapeutic activity *in vivo* after i.v. injection in B16 melanoma-bearing mice. It must be remembered, however, that this study has been performed with human LDL. Even if human LDL is recognised by the animal's receptor and particularly by the B16 melanoma tumour (Lombardi *et al.*, 1988), the competition from endogenous LDL is very different from the one in the human situation since there are pronounced species differences in the plasma lipoprotein pattern and metabolism. Therefore, the results must be interpreted with caution. It would be suitable to use an animal model close to the human one for the lipoprotein metabolism such as hamster or guinea pig.

Interestingly, daily fractional doses are more effective than single injection. This could be explained, partly by the fact that the uptake of LDL by the receptor pathway is a saturable process and partly by the fact that a high level of LDL cholesterol could down-regulate the expression of the LDL receptor (Brown & Goldstein, 1986). These data suggest that administration of drug complex as an infusion rather than a bolus could be preferable for a greater efficiency.

We use a technique which allows a high entrapment level of lipophilic cytotoxic drugs into the LDL (this technique may be applied to other lipophilic compounds, unpublished data). The cytotoxic drug-LDL complex so formed, exhibits preferentially LDL receptor related catabolism *in vivo* like native LDL, and increasing antitumour activity in a murine tumour model that expresses high LDL uptake by the tumour. Further human clinical trials will depend on a better knowledge of the expression and the regulation of the LDL receptor by the tumour cells; which histological cell type, which growth or transformation stage, etc. is involved. Studies in this optic are now in progress in our laboratory.

This work was supported in part by: 'La Fédération Nationale des Centres de Lutte Contre le Cancer, les Comités Départementaux de la Région Midi-Pyrénées, la Ligue Nationale de Lutte Contre le Cancer' and by 'Agence Nationale de Valorisation et d'Aide à la Recherche, ANVAR'.

We would like to thank Françoise Cheutin for technical assistance.

**Abbreviations:** 9-OH-NME, 9-hydroxy-$N^2$-methyl ellipticinium acetate; OL-NME, 9-oleoyloxy-$N^2$-methyl ellipticinium oleate; LDL, low density lipoprotein; VLDL, very low density lipoprotein; HDL, high density lipoprotein; LPDS, lipoprotein-deficient serum; HPLC, high-performance liquid chromatography; TG, triacyl glycerol; PC, phosphatidyl choline; PS, phosphatidyl serine; Sphin, spingomyelin; Ch, cholesterol; BSA, bovine serum albumin; i.v., intravenous; i.p., intraperitoneal; s.c., subcutaneous.

### References

AUCLAIR, C., PIERRE, A., VOISIN, E., PEPIN, O., CROS, S., COLAS, SAUCIER, J.M., VERSCHUERE, B., GROS, P. & PAOLETTI, C. (1987). Physiochemical and pharmacological properties of the antitumour ellipticine derivative 2-(diethylamino-2-ethyl) 9-hydroxy ellipticinium-chloride, HCl. *Cancer Res.*, **47**, 6254–6261.

AVIRAM, M., BIERMAN, E.L. & CHAIT, A. (1988). Modification of low density lipoprotein by lipoprotein lipase or hepatic lipase induces enhanced uptake and cholesterol accumulation in cells. *J. Biol. Chem.*, **263**, 15416–15422.

BILHEIMER, O.H., EISENBERG, S. & LEVY, R.I. (1972). The metabolism of very low density lipoprotein. I. Preliminary *in vitro* and *in vivo* observation. *Biochem. Biophys. Acta.*, **260**, 212–221.

BROWN, M.S. & GOSDSTEIN, J.L. (1986). A receptor-mediated pathway for cholesterol homeostasis. *Sciences*, **232**, 34–47.

CHAIT, A., EISENBERG, S., STEINMETZ, A., ALBERS, J.J. & BIERMAN, E.L. (1984). Low density lipoprotein modified by lipid transfer protein have altered biological activity. *Biochem. Biophys. Acta.*, **795**, 314–325.

DE SMIDT, P.C. & VAN BERKEL, T.J.C. (1990). Prolonged serum half-life of antineoplastic drugs by incorporation into the low density lipoprotein. *Cancer Res.*, **50**, 7476–7482.

DULLAART, R.P.F., GROENER, J.E.M. & ERKELENS, D.W. (1987). Effect of the composition of very low density lipoproteins on the rate of cholesteryl ester transfer from high density lipoproteins in man, studied *in vitro*. *Eur. J. Clin. Invest.*, **17**, 241–248.

EISENBERG, S. (1985). Preferential enrichment of large sized very low density lipoprotein population with transferred cholesteryl esters. *J. Lipid. Res.*, **26**, 487–494.

GAL, D., OTTASHI, M., MACDONALD, P.C., BUSCHBAUM, H.J. & SIMPSON, E.R. (1981). Low-density lipoprotein as a potential vehicle for chemotherapeutic agents and radionucleotides in the management of gynecologic neoplasms. *Am. J. Obstest. Gynecol.*, **139**, 877–885.

GOLDSTEIN, J.L. & BROWN, M.S. (1977). The low density lipoprotein pathway and its relation to atherosclerosis. *Annual Reviews in Biochemistry*, **46**, 897–930.

GOLDSTEIN, J.L. & BROWN, M.S. (1974). Binding and degradation of low density lipoproteins by cultured human fibroblasts. *J. Biol. Chem.*, **246**, 5153–5162.

GOTTO, A.M. Jr, POWNALL, H.J. & HAVEL, R.J. (1986). Introduction to the plasma lipoproteins. In Segrest, J.P. & Albers, J.J. (eds) *Methods in Enzymology*, vol. 128. Olando, FL: Academic Press 3-41.

HAVEL, R.J., EDER, M.A. & BRAGDON, J.H. (1955). The distribution and chemical composition of ultracentrifugally separate lipoproteins in human plasma. *J. Clin. Invest.*, **34**, 1345–1350.

HYNDS, S.A., WELSH, J., STEWART, M.J., JACK, A., SOUKOP, M., MACARDLE, C.S., CALMAN, K.C., PACKARD, C.J. & SHEPHERD, J. (1984). Low-density lipoprotein metabolism in mice with soft tissue tumours. *Biochim. Biophys. Acta.*, **795**, 589–595.

IWANIK, M.J., SHAW, K.V., LEDWITH, B.J., YANOVICH, S. & SHAW, J.M. (1984). Preparation and interaction of a low-density lipoprotein: daunomycin complex with P388 leukemic cells. *Cancer Res.*, **44**, 1206–1215.

KLEINMANY, Y. (1987). Hypolipidemic therapy modulates expression of apolipoprotein B epitopes on low density lipoproteins. Studies in mid to moderate hypertriglyceridemic patients. *J. Lipid. Res.*, **28**, 540–548.

KODAMA, T., FREEMAN, M., ROHRER, L., ZABRECKY, J., MATSUDAIRA, P. & KRIEGER, M. (1990). Type I macrophage scavenger receptor contains – helical and collagen-like coiled coils. *Nature*, **343**, 531–535.

KRIEGER, M., SMITH, L.C., ANDERSSON, R.G., GOLDSTEIN, J.L., KAO, Y.J., POWNALL, H.J., GOTTO, A.M.Jr & BROWN, M.S. (1979). Reconstituted low density lipoprotein: a vehicle for the delivery of hydrophobic fluorescent probes to cells. *J. Supramol. Structure*, **10**, 467–470.

LESTAVEL-DELATTRE, S., MARTIN-NIZARD, F., CLAVEY, V., TESTARD, P., FAVRE, G., DOUALIN, G., SQALLIHOUSSAINI, H., BARD, J.M., DURIEZ, P., DELBART, C., SOULA, G., LESIEUR, D., LESIEUR, I., CAZIN, J.C., CAZIN, M. & FRUCHART, J.C. (1992). Low-density lipoprotein for delivery of an acrylophenone antineoplastic molecule into malignant cells. *Cancer Res.*, **52**, 3629–3635.

LOMBARDI, P., NORATA, G., MAGGI, F.M., CANTI, G., FRANCO, P., NICOLIN, A. & CATAPANO, A.L. (1988). Assimilation of LDL by experimental tumours in mice. *Biochim. Biophys. Acta*, **1003**, 301–306.

LOWRY, O.H., ROSEBROUGH, N.J., FARR, A.L. & RANDALL, R.J. (1951). Protein measurement with the folin phenol reagent. *J. Biol. Chem.*, **193**, 265–275.

MAHLEY, R.W., INNERARITY, T.L., PITAS, R.E., WEISGRABER, K.H., BROWN, J.H. & GROSS, E. (1977). Inhibition of lipoprotein binding to cell surface receptors of fibroblasts following selective modification of arginyl residues in arginine-rich and B. apoprotein. *J. Biol. Chem.*, **252**, 7279–7287.

MASQUELIER, M., VITOLS, S. & PETERSON, C. (1986). Low-density lipoprotein as a carrier of antitumoural drugs: *in vivo* fate of drug-human low-density lipoprotein complexes in mice. *Cancer Res.*, **46**, 3842–3847.

MAYER, L.D., BALLY, M.B., LOUGHREY, H., MASIN, D. & CULLIS, P.R. (1990). Liposomal vincristine preparations which exhibit decreased drug toxicity and increased activity against murine L1210 and P388 tumours. *Cancer Res.*, **50**, 575–579.

MAZZONE, T., BASHEERDING, K., PING, L. & SCHICK, C. (1990). Relation to growth and sterol regulatory pathway for low density lipoprotein receptor gene expression. *J. Biol. Chem.*, **265**, 5145–5149.

MILLER, K.W. & SMALL, D.M. (1982). The phase behaviour of triolein, cholesterol and lecithin emulsions. *J. Coll. Interface Sci.*, **89**, 466–478.

MORTON, R.E. & ZILVERSMIT, D.B. (1983). Interrelationship of lipids transferred by the lipid transfer protein isolated from human lipoprotein deficient plasma. *J. Biol. Chem.*, **258**, 1751–1757.

PAOLETTI, C., LE PECQ, J.B., DAT-XUONG, N., JURET, P., GARNIER, H., AMIEL, J.L. & ROUESSE, J. (1980). Antitumour activity pharmacology and toxicity of ellipticinium and 9-hydroxy derivatives: preliminary clinical trials of 2-methyl-9-hydroxyellipticinium (NSC 264137). *Cancer Res.*, **74**, 107–123.

PARKS, J.S., MARTIN, J.A., JOHNSON, F.L. & RUDEL, L.L. (1985). Fusion of low density lipoproteins with cholesterol ester-phospholipid micro emulsions. *J. Biol. Chem.*, **260**, 3155–3163.

PATSCH, J.R., SAILER, S., KOSTNER, G., SANDHOFER, F., HOLASEK, A. & BRAUNSTEINER, H. (1974). Separation of the main lipoprotein classes from human plasma by rate-zonal ultracentrifugation. *J. Lipid Res.*, **15**, 356–360.

PAUMAY, Y. & RONVEAUX-DUPAL, M.F. (1985). Rapid preparative isolation of concentrated low density lipoprotein and of lipoprotein-deficient serum using vertical rotor gradient ultracentrifugation. *J. Lipid Res.*, **26**, 1476–1479.

RUBEN, R.L. & NEUBAUER, R.H. (1987). Semiautomatic colorimetric assay for *in vitro* screening of anticancer compounds. *Cancer Treat. Rep.*, **71**, 1141–1149.

RUDLING, M.J., REIHNER, E., EINARSSON, K., EWERTH, S. & ANGELIN, B. (1990). Low density lipoprotein receptor-binding activity in human tissues: quantitative importance of hepatic receptors and evidence for regulation of their expression *in vivo*. *Proc. Natl Acad. Sci. USA*, **87**, 3469–3473.

SAMADI-BABOLI, M., FAVRE, G., BERNADOU, J., BERG, D. & SOULA, G. (1990). Comparative study of the incorporation of ellipticin-esters into Low Density Lipoprotein (LDL) and selective cell uptake of drug-LDL complex via the LDL receptor pathway *in vitro*. *Biochem. Pharmacol.*, **40**, 203–212.

SAMADI-BABOLI, M., FAVRE, G., BLANCY, E. & SOULA, G. (1989). Preparation of low density lipoprotein-9-methoxy ellipticin complex and its cytotoxic effect against L1210 and P388 leukemic cells *in vitro*. *Eur. J. Cancer Clin. Oncol.*, **25**, 233–241.

SHAW, J.M., SHAW, K.V., YANOVICH, S., IWANIK, M. & FUTCH, W.S. (1988). Delivery of lipophilic drugs using lipoproteins. *Ann. NY Acad. Sci. USA*, **507**, 252–271.

SHEPHERD, J., BICKER, S., ROSS LORIMER, A. & PACKARD, C.J. (1979). Receptor-mediated low density lipoprotein catabolism in man. *J. Lipid. Res.*, **20**, 999–1006.

SHEPHERD, J., PACKARD, C.J., BICKER, S., LAWRIE, V. & MORGAN, H.G. (1980). Cholesterylamine promotes receptor-mediated low-density-lipoprotein catabolism. *N. Engl. J. Med.*, **302**, 1219–1222.

SLATER, H.R., PACKARD, C.J. & SHEPHERD, J. (1982). Measurement of receptor-independent lipoprotein catabolism using 1,2 cyclohexadione-modified low density lipoprotein. *J. Lipid. Res.*, **23**, 92–96.

VITOLS, S., ANGELIN, B., ERICSSON, S., GAHRTON, G., JULIOSSON, G., MASQUELIER, M., PAUL, C., PETERSON, C., RUDLING, M., SODERBERG-REID, K. & TIDEFELDT, U. (1990*a*). Uptake of low density lipoproteins by human leukemic cells *in vivo*: relation to plasma lipoprotein levels and possible relevance for selective chemotherapy. *Proc. Natl Acad. Sci. USA*, **87**, 2598–2602.

VITOLS, S., GAHRTON, G., BJORKHOLM, M. & PETERSON, C. (1985). Hypocholesterolaemia in malignancy due to elevated low-density-lipoprotein-receptor activity in tumour cells: evidence from studies in patients with leukaemia. *Lancet*, **ii**, 1150–1154.

VITOLS, S., SODERBERG-REID, K., MASQUELIER, M., SJOSTROM, B. & PETERSON, C. (1990*b*). Low density lipoprotein for delivery of a water-insoluble alkylating agent to malignant cells. *In vitro* and *in vivo* studies of a drug-lipoprotein complex. *Br. J. Cancer*, **62**, 724–729.